# The Work of the Metropolitan Asylums Board.—III

**Published:** 1902-01-18

**Authors:** 


### The Institutional Workshop.

### THE WORK OF THE METROPOLITAN ASYLUMS BOARD.—III.

By the Author of "The Hospital Commissariat."

The boundaries of the Asylums District are practically the boundaries of Greater London. It extends from Harringay Park in the north to a line just excluding the Crystal Palace in the south; from Acton and Hammersmith (excluding Barnes) on the west to Poplar, Plumstead, and Eltham on the east.

This wide area is mapped out into districts and is served by ten fever hospitals, for the most part named, on rather a bewildering plan, after the points of the compass. Thus there are Eastern, North-eastern and South-eastern; Western, North-western and South-western fever hospitals. In addition there are two gigantic establishments at Tooting, known as the Grove and the Fountain Hospitals; the Park Hospital at Hither Green, S.E., and the Brook Hospital at Shooter's Hill. In all the Metropolis makes up a total of 4,419 beds for acute fever cases.

Experience has shown that the patient must be retained in isolation long after he is practically cured, and the public safety is secured no less by the institutions for convalescing patients than by those for acute cases. It is probable that all the earlier attempts at isolation hospitals failed largely because the authorities failed fully to appreciate the danger to the public of the convalescent. The Northern Hospital at Winchmore Hill can receive 764 fever convalescents, and a similar establishment, to be known as the Southern Hospital, is in course of erection at Carshalton for the reception of 800 more.

The accommodation provided by the Board for small-pox patients consists at present of 300 beds for acute cases in the hospital ships, and 1,192 beds for convalescing patients at Gore Farm. It is evident that the hospital ships are quite inadequate in accommodation to the strain of even a moderate epidemic of small-pox, which may at any time, spite of all precautions, have to be encountered. The Board has therefore acquired land in the neighbourhood of Dartford, Kent, for the erection of a building for small-pox patients to contain 400 beds.

The maintenance of enormous buildings in a high state of efficiency, equipped with a permanent staff and ready for any emergency, yet practically unoccupied from one year's end to another, is one of the remarkable features of London's bulwarks against infection. Municipal authorities and charitable agencies in all the ages past have rebelled against the apparent wastefulness of such foresight, and no epidemic that has ever laid waste the homes of the people has till now been encountered by an expert organisation, quietly watchful for its beginnings and of strength to grapple with it at its height.

The ambulance work of the Board is a very important addition to the protection of the public from infection. There are now six ambulance stations—Eastern, South-eastern, Western, South-western, North-western and Brook—and every year the conveyance work of the department increases. A few years ago the only course open to a fever or small-pox patient who must be removed from crowded surroundings was to engage at a high price some hired conveyance, which might or might not be partially disinfected afterwards, and the part played by four-wheeled cabs in the dissemination of disease was probably a very large one. In 1899 the ambulances of the Board, besides conveying over 40,000 patients to and fro in connection with their own hospitals, carried over 1,000 fever cases in connection with the general hospitals and to other destinations.

The modern ambulance bears no resemblance to the gloomy-looking death-traps which may be remembered by some as the early form of conveyance for the sick. The carriages are equipped for one or two patients; they contain berths on which the patient may lie in comfort, and are well ventilated and lighted. A nurse is always in attendance. The ambulance stations have been doubled in number since the establishment of the service, and have increased in importance, till they have become one of the most valuable factors in the Board's protective work.

The concentration of fever patients in special hospitals considerably lessened the chances of the medical student for getting any clinical instruction during his medical education in this class of case. And as at the same time the hospitals of the Board grew every year more efficient, it became evident that a great waste of teaching material was taking place. This was fully recognised by the Local Government Board in 1890, when the Asylums Board Hospitals were opened to medical students after the completion of their third year, and to qualified practitioners generally. The minimum course of study for which a certificate could be granted was fixed at two months, attendance being expected at least twice a week, and the fee for this period was three guineas. Two years later a further order of the Local Government Board introduced a separate set of regulations for study in the small-pox hospitals of the Board. The course was fixed at a minimum of two weeks, during which time the student was expected to reside on the premises, entirely under the jurisdiction of the medical superintendent, and paying a small weekly sum for his board in addition to a fee of two guineas for the course. The number of students who take advantage of the opportunities offered is not large, but is increasing. In 1899, 459 male students and 25 female students attended classes at the Boards' fever hospitals. Opportunities for gaining clinical instruction in small-pox are practically non-existent elsewhere.

Among the labours of the Asylums Board must not be forgotten the compiling of elaborate and very careful statistics relating to the various outbreaks of fever which occur in the Metropolis. Maps are issued annually, showing by spots the position of every case of diphtheria, scarlet-fever, typhus or enteric in the Metropolis during the four quarters of the year. This renders it possible to concentrate attention immediately on the sanitary conditions of any locality where such spots recur with any regularity, and places the whole of London under direct and continuous observation. It has even become possible by means of highly trained observation and deduction to forecast with almost magical accuracy, months in advance, the probable demands which will be made on the Board for accommodation in the fever hospitals. Thus we read: "On July 5th (1899) the Hospitals Committee were advised that the maximum number of beds which would probably be required in the ensuing autumn for scarlet-fever would be about 3,600, and for diphtheria about 1,800. The maximum numbers actually attained were: Scarlet-fever cases 3,535 (on October 5th), and diphtheria cases 1,768 (on November 23rd)."

One word, in conclusion, as to the expense entailed by this great system of municipal watchfulness. In 1899 the total cost of the hospitals of the Board and all expenses connected with them amounted to £340,000. To this must be added an expenditure of £28,700 for the ambulance service, making a total of £368,700. It is a large sum. But were it possible to make any computation of the amount of money saved to individuals by the immediate removal of infectious patients from their surroundings, it would undoubtedly be found that, putting all other considerations aside, in point of economy alone the hospitals of the Metropolitan Asylums Board are doing a great work for London.

